# Individualized detection of TMPRSS2-ERG fusion status in prostate cancer: a rank-based qualitative transcriptome signature

**DOI:** 10.1186/s12957-024-03314-8

**Published:** 2024-02-09

**Authors:** Yawei Li, Hang Su, Kaidong Liu, Zhangxiang Zhao, Yuquan Wang, Bo Chen, Jie Xia, Huating Yuan, De-Shuang Huang, Yunyan Gu

**Affiliations:** 1 School of Biology and Engineering, Guizhou Medical University, Guiyang, Guizhou, China; 2 School of Clinical Medicine, Guizhou Medical University, Guiyang, Guizhou, China; 3 College of Bioinformatics Science and Technology, Harbin Medical University, Harbin, Heilongjiang, China; 4 The Sino-Russian Medical Research Center of Jinan University, The Institute of Chronic Disease of Jinan University, The First Affiliated Hospital of Jinan University, Guangzhou, China; 5 Bioinformatics and BioMedical Bigdata Mining Laboratory, School of Big Health, Guizhou Medical University, Guiyang, Guizhou, China

**Keywords:** Prostate cancer, Qualitative signature, TMPRSS2-ERG fusion, 5-ERG-mRPs, Cross-platform

## Abstract

**Background:**

TMPRSS2-ERG (T2E) fusion is highly related to aggressive clinical features in prostate cancer (PC), which guides individual therapy. However, current fusion prediction tools lacked enough accuracy and biomarkers were unable to be applied to individuals across different platforms due to their quantitative nature. This study aims to identify a transcriptome signature to detect the T2E fusion status of PC at the individual level.

**Methods:**

Based on 272 high-throughput mRNA expression profiles from the Sboner dataset, we developed a rank-based algorithm to identify a qualitative signature to detect T2E fusion in PC. The signature was validated in 1223 samples from three external datasets (Setlur, Clarissa, and TCGA).

**Results:**

A signature, composed of five mRNAs coupled to ERG (five ERG-mRNA pairs, 5-ERG-mRPs), was developed to distinguish T2E fusion status in PC. 5-ERG-mRPs reached 84.56% accuracy in Sboner dataset, which was verified in Setlur dataset (*n* = 455, accuracy = 82.20%) and Clarissa dataset (*n* = 118, accuracy = 81.36%). Besides, for 495 samples from TCGA, two subtypes classified by 5-ERG-mRPs showed a higher level of significance in various T2E fusion features than subtypes obtained through current fusion prediction tools, such as STAR-Fusion.

**Conclusions:**

Overall, 5-ERG-mRPs can robustly detect T2E fusion in PC at the individual level, which can be used on any gene measurement platform without specific normalization procedures. Hence, 5-ERG-mRPs may serve as an auxiliary tool for PC patient management.

**Supplementary Information:**

The online version contains supplementary material available at 10.1186/s12957-024-03314-8.

## Introduction

Prostate cancer (PC) is the major public health challenge for men, with an estimated 288,300 new cases diagnosed and 34,700 deaths in 2023 from the disease in the USA [1]. Due to the high death rate of PC, many studies have been conducted to improve the prognosis of PC. For instance, Montazersaheb et al. have proved for the first time that betanin, when used in combination with radiotherapy, contributes to cytotoxic and apoptotic effects on PC cells [2]. Identifying potential drugs to disease and exploring ways to increase sensitivity to drug therapy were also effective strategy for extending the survival time of cancer patients [3–5]. Besides, genomic events impact patient outcomes. Tomlin et al. reported the most common form of PC-specific fusion with more than 50% occurrence frequency, which is TMPRSS2-ERG (T2E) fusion [6]. T2E fusion is mainly produced by intermediate deletion and balanced chromosome translocation. The former refers to the deletion in the middle part of the chromosome arm, and the latter refers to the position change after the breakage of two chromosomes [7]. This event caused the oncogenic effects of aberrant transcription factors, specifically the overexpression of *ERG*, which plays a critical role in the regulation of cell growth, differentiation, and apoptosis [8]. Thus, T2E fusion is associated with a more aggressive clinical presentation of PC. Besides, a recent discovery indicated that T2E fusion is an early event in PC and could provide support for PC diagnosis [9].

Gene fusions driven by chromosomal translocations are highly correlated with patient clinical outcomes [10]. Numerous studies have demonstrated that T2E fusion is a risk factor in PC [11–13]. Therefore, the detection of T2E fusion has implications for prognosis prediction and postoperative management of PC patients. Currently, there are three primary approaches for determining the T2E fusion status in PC, involving methods based on fluorescence in situ hybridization (FISH) or immunohistochemistry (IHC), high-throughput sequencing technology, and transcriptional signatures. Despite the high sensitivity (> 80%) of FISH and IHC, sample fixation techniques, reference settings, and reagent batches all have an impact on the results [14, 15]. Besides, semi-quantitative and subjective positive interpretation criteria also lead to poor reproducibility of detection results between laboratories. Based on RNA sequencing data, researchers had developed many tools for identifying gene fusion, but the criteria for screening candidate fusion genes and filtering out false positive reports are different among tools. The detection accuracy of these tools is highly sensitive to the length of reads, with a 40–90% area under the curve (AUC) [15]. Many transcriptional signatures were also identified for the detection of T2E fusion [16–18]. However, the application of these signatures depends on the conversion score of the absolute expression measurements of signature genes, which are affected by systematic measurement bias induced by experimental batch effects [19]. In contrast, relative expression orderings (REOs) of gene pairs can overcome these limitations and be applied to individual samples without a complex scoring process. Besides, our previous study showed that REO-based biomarkers are relatively robust against poor sample quality [20–22].

In this study, using gene quantitative expression of PC from Gene Expression Omnibus (GEO) and The Cancer Genome Atlas (TCGA) database, we identified a qualitative REO-based signature composed of five gene pairs for the detection of T2E fusion status of PC. The signature was tested in three independent datasets and compared with four fusion prediction tools based on several T2E fusion-related features.

## Materials and methods

### Bulk data and preprocessing

We conducted an electronic search without limitations on publication date in PubMed and GEO (Gene Expression Omnibus) to find all articles and collecting gene expression datasets reporting on signature and/or biomarker in PC related to T2E fusion. Four PC-related gene expression datasets were then used in this study, which were downloaded on December 6, 2022, from the GEO (http://www.ncbi.nlm.nih.gov/geo/), TCGA (http://cancergenome.nih.gov/), and Cbioportal (http://www.cbioportal.org/). As described briefly in Table 1, the Sboner dataset was considered the training dataset for extracting the rank-based T2E fusion signature. Three testing datasets included 455 samples (Setlur dataset) generated by Human 6 k Transcriptionally Informative Gene Panel for DASL, 495 samples (TCGA dataset) generated by Illumina Hiseq RNAseqV2, and 118 samples (Clarissa dataset) generated by RNA-seq, respectively.

**Table 1:** The datasets analyzed in this study

Dataset	Accession	TFP	TFN	Platform
Sboner, 2010	GSE16560	46	226	Human 6 K for DASL
Setlur, 2008	GSE8402	103	352	Human 6 K for DASL
TCGA	TCGA	–	–	Illumina RNA-seqV2
Clarissa, 2018	DKFZ	86	32	RNA-seq

*TFP*, T2E fusion-positive, *TFN*, T2E fusion-negative

For the Sboner and Setlur datasets derived from GEO, the transcriptome expression profile was extracted from “series.txt” files. The same preprocessing was performed, wherein the probe-set identifiers (IDs) were mapped to Entrez gene IDs using the corresponding platform files. For each sample, the expression measurements of multiple probe-set mapped to the same Entrez gene ID were averaged to obtain a single measurement, and probe-sets that did not map any gene ID or mapped multiple gene IDs were deleted. For dataset TCGA, the mRNA-seq profiles of level 3 Fragments Per Kilobase Million were extracted. The mRNA expression (RNA seq RPKM) was used as the gene expression measurements of the Clarissa dataset.

### Single-cell data quality control and analysis

Single-cell data of six PRAD tumor samples, the Dong dataset, were obtained from Dong et al. [23]. R package “seurat” (V4.0.5) was performed for data preprocessing and subsequent analysis [24]. All functions were run with default parameters. To filter low-quality cells, only cells that have unique feature counts less than 2500, or over 200, and that have < 5% mitochondrial counts were included in the following analysis. Identification of cell population was performed using marker genes achieved from a recent publication [25] and summarized in Table S1. The cells were predicted as tumor cells by Copycat method and whose cell types were annotated as epithelial cells or endothelial cells were regarded as tumor cells [26]. For *N* tumor cells from sample* s*, we extracted randomly *n* cells without replacement (*n* = 1, 2, …, *N*), and *N* bulk tissues were obtained. For one sampling, the mean expression level of these *n* cells represents the gene expression profile of this bulk tissue. Then, based on the binomial distribution test, sample *s* was classified into T2E fusion-positive (TFP) or T2E fusion-negative (TFN) group if significantly more bulk tissues were classified into TFP or TFN group by our signature.

Identification of *ERG* paired genes.

Based on the *F*-statistic from the one-way ANOVA test, Shannon’s entropy, coefficient of variation (CV), the outlier sum statistic, and the median absolute deviation (MAD) of the expression of genes, we calculated five metrics of variability for genes in the training dataset. *F*-statistic was used to assess the differences in gene expression between TFP and TFN tumors. Shannon’s entropy, considered as a measure of information content for the quantitative expression of genes, was computed using entropy R package. CV was computed as ${\text{CV}}=\frac{\sigma }{\mu }$, where *σ* is the overall standard deviation of the expression level of a gene in all samples and *µ* is the average expression level. The outlier sum statistic, a variability for identifying the presence of outliers, was calculated by the sum of absolute value from median-centered outliers. Here, outliers were detected by IQR (interquartile range) method that the quantitative expression measurements of genes fall outside Q1 − IQR or Q3 + IQR. MAD was calculated as ${\text{MAD}}={\text{median}}(|{E}_{i}-{\text{median}}({E}_{i})|)$, where *E*_*i*_ represents the expression values of gene *i.* The smaller each variable, the more stable the expression level of gene between TFP and TFN tumors. Further, the stingscore method was used to integrate these five variabilities [27]. The genes with scores in the top quartile (unstable) and bottom quartile (stable) were defined as *ERG*-paired genes.

### Development of the T2E signature

Firstly, we computed the reverse degree of the combination of *ERG* and reference genes, which is defined as follows:

$${\overline{R} }_{(ERG, Ref)}=\sqrt{\frac{|{\sum }_{1}^{i}\left({R}_{ERG[{N}_{i}]}-{R}_{Ref[{N}_{i}]}\right)|}{m}\times \frac{|{\sum }_{1}^{j}\left({R}_{ERG[{P}_{j}]}-{R}_{Ref[{P}_{j}]}\right)|}{n}}$$

where ${R}_{ERG[{N}_{i}]}$ and ${R}_{Ref[{N}_{i}]}$ represented the rank of gene expression measurements of *ERG* and reference genes in TFN samples, respectively ($i$ = 1…$m$. $m$ is the sample size of TFN samples). Similarly, ${R}_{ERG[{P}_{j}]}$ and ${R}_{Ref[{P}_{j}]}$ represented the rank of *ERG* and reference genes in TFP samples ($j$ = 1…$n$. $n$ is the sample size of TFP samples), respectively. Then, gene pairs with the top 20% ${\overline{R} }_{(ERG, Ref)}$ were defined as gene pairs with high reversion degree. Secondly, the quantitative expression of *ERG* and reference genes were transformed into the within-sample REOs of gene pairs (*E*_*ERG*_ > *E*_*reference*_ or *E*_*ERG*_ < *E*_*reference*_). Samples were classified into the TFN or TFP groups for each ERG-reference gene pair according to their within-sample REO pattern. Then, the F-1 values for each gene pair were evaluated by computing the sample size of correct classifications, which was calculated as follows:

$${\text{F}}-1=\frac{2\times ({Sen}_{(ERG, Ref)}\times {Spe}_{(ERG, Ref)})}{{Sen}_{(ERG, Ref)}+{Spe}_{(ERG, Ref)}}$$

Gene pairs with the top 20% F-1 value were termed as gene pairs with high discrimination degree. Finally, the top 10 gene pairs from gene pairs with high reversion degree or high discrimination degree and the overlapped between them were defined as candidate gene pairs. Finally, from these candidate gene pairs, we performed a forward selection procedure to discovery T2E signature that achieved the largest F-1 value based on the pre-defined classification rule: a sample was classified into the TFP subtype if at least half of the gene pairs within this sample vote for TFP; otherwise, the TFN subtype. Among the results derived from the seeds with top 10 F-1 values, a set of gene pairs with the highest F-1 value was chosen as the T2E signature.

### ssGSEA scores of oncogenic, metabolism pathways and immune cell infiltration

The ssGSEA method was used to quantify the fifty oncogenic pathways activity (http://www.broadinstitute.org/msigdb, h.all.v7.4.entrez.gmt) [28]. Further, the immune infiltration of 28 immune cell types was also evaluated by ssGSEA method, whose signature genes were derived from literature [29]. The fraction of M1 macrophage and M2 macrophage, the immune score, and stromal score of 435 PC samples in TCGA by xCell method were downloaded from the TCGA immune-related database (http://timer.cistrome.org/). The immune score and stromal score of these samples also computed by the Estimate method using R package “estimate.” Besides, cytolytic (CYT) score of these PC samples was derived from a recent research [30]. The one-sided Wilcoxon rank-sum test was used to compare the immunity features mentioned above and the ssGSEA score of pathways between the two groups.

### Survival analysis

The overall survival (OS) was termed as the time from the date of initial surgical resection to death or last contact (censored). The survival status of patients beyond 10 years was transformed to dead; OS was changed to 10 years. Survival curves of OS were estimated using the Kaplan–Meier method and were compared using the log-rank test [31]. The multivariate Cox proportional-hazards regression model was introduced to evaluate the independent prognostic value of the signature after adjusting for clinical factors including Gleason score, age, PSA level, and stage.

### L1000 drug connectivity analysis

Limma was performed to determine differential abundance of genes between TFP and TFN tumors. Genes with FDR < 0.01 were considered as differentially expressed. With a dysregulation frequency cutoff of 80%, the universal differentially expressed genes (DEGs) were detected by RankComp V1 at least in one of TCGA and PRAD_DKFZ datasets [32]. Using Spearman’s rank correlation (|*r*|> 0.6, FDR < 0.01), the universal DEGs that were associated with the ssGSEA score of one of fifty oncogenic pathways were then used as inputs in the subsequent analysis. Input genes were measured in the L1000 gene panel and then were processed using connectivity map (cMAP) tool (https://clue.io/). The resulting drug connectivities were aggregated to the compound level using the raw connectivity score in cMAP. Target annotations for the ranked compounds were extracted from cMAP. The directionality of the connectivity score (positive or negative) represented the effects of drugs (antagonistic effects or synergistic effects).

### Statistical analysis

Since evidence has demonstrated that T2E fusion is a risk factor for PC [11–13], the samples in our study possessing the attribute of T2E fusion-positive are called the “case,” and those without it are the “control.” All statistical analyses in this study were carried out using R software version 4.0.2 (http://www.r-project.org/).

## Result

### Identifying and validation of T2E fusion qualitative signature

**Fig. 1:**
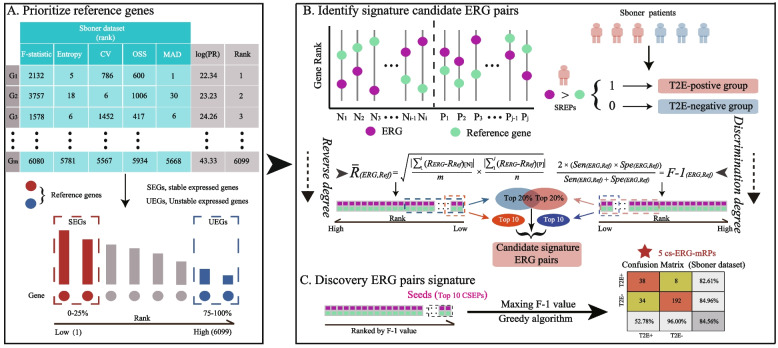
Workflow of this study. **A** Prioritizing reference genes based on five metrics of variability for genes by stingscore method. **B** Identifying signature candidate gene pairs combined with *ERG* based on reverse and discrimination degrees of gene pairs. **C** A qualitative signature consisting of 5 gene pairs was developed by performing a forward selection approach, denoted as 5-ERG-mRPs

**Fig. 2:**
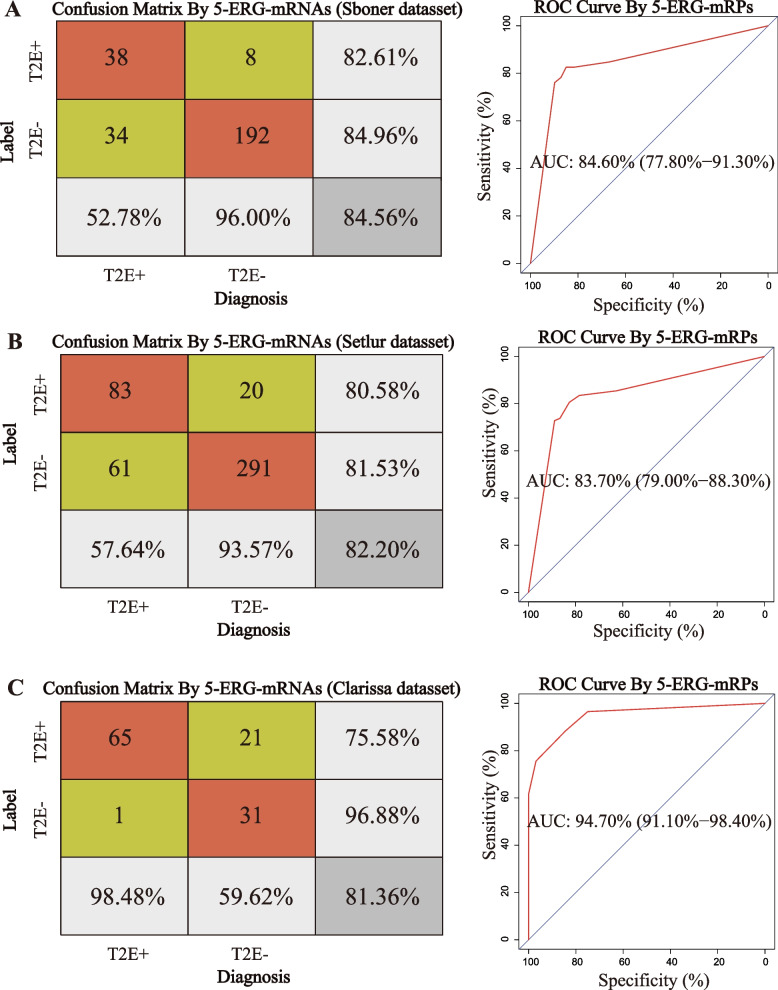
Performance of 5-ERG-mRPs in the training and validation datasets. **A** Confusion matrix and ROC curve for the Sboner dataset by 5-ERG-mRPs. **B** Confusion matrix and ROC curve for the Setlur dataset by 5-ERG-mRPs. **C** Confusion matrix and ROC curve for the Clarissa dataset by 5-ERG-mRPs

The reference genes were denoted as stably expressed genes (SEGs) or unstable expressed genes (USEGs) between TFP and TFN tumors. Then, based on the evidence that the elevated expression of *ERG* would be caused by T2E fusion, the REO pattern of pairwise ERG-SEGs or ERG-USEGs might be reversed in TFP tumors compared with TFN tumors. Therefore, a procedure of identifying T2E fusion signature was constructed (Fig. 1). Firstly, according to gene expression variation between tumors with different T2E fusion statuses in the training dataset (*n* = 272), 3050 genes ranked top or bottom quartiles were identified as the reference genes by a rank fusion method with five indexes (Fig. 1A). Figure S1 shows distributions of these measurements in the Sboner dataset. Secondly, from the 3050 ERG-reference pairs, 55 gene pairs with a high reverse degree and/or high discrimination degree were retained according to a filtering rule in “Materials and methods” (Fig. 1B). Finally, based on the 55 candidate gene pairs, a set of five gene pairs with the highest reverse degree and discrimination degree (F-1 value = 0.8377) were selected as the T2E fusion signature by using greedy method, denoted as 5 ERG-mRNA pairs (5-ERG-mRPs) (Fig. 1C). With the majority voting rule, 38 of 46 TFP samples and 192 of 226 TFN samples in the training dataset were correctly classified. The accuracy and AUC of 5-ERG-mRPs were 84.56% and 84.60% (95% CI: 77.80–91.30%, Fig. 2A). Besides, as shown in Fig. S2A, when stratifying patients based on the median expression value of *ERG*, though the AUC reached 82.50% (95% CI: 74.90–90.10%), the accuracy of *ERG* was only 66.18%.

Then, 5-ERG-mRPs was applied in two independent datasets. For the 455 samples in the Setlur dataset, 5-ERG-mRPs resulted in a diagnosis of T2E fusion status with an 80.58% sensitivity (83/103), 81.53% specificity (291/352), 82.20% accuracy (374/455), and 83.70% AUC (95% CI: 79.00–88.30%, Fig. 2B). For the 118 samples in the Clarissa dataset, 65 of 86 TFP samples and 31 of 32 TFN samples were correctly classified by 5-ERG-mRPs; the AUC reached 94.70% (95% CI: 91.10–98.40%, Fig. 2C). We validated the performance of the *ERG* in the two datasets; the accuracy was decreased to 63.74% and 77.12% in the Setlur and Clarissa datasets, respectively (Fig. S2B, C).

### The classification performance of 5-ERG-mRPs is better than fusion prediction tools

Comparison analysis was then conducted in the dataset TCGA between 5-ERG-mRPs and current fusion prediction tools, including PRADA (TumorFusion), Tophat-Fusion, STAR-Fusion, and TCGA FAWG. For 495 samples in TCGA, 179 and 316 samples were classified by 5-ERG-mRPs into TFP and TFN groups, respectively. The results of fusion prediction tools were obtained for dataset TCGA from ChimerDB [33]. As shown in Fig. S3, 435 samples were predicted consistently by signature and fusion prediction tools, denoted as reference samples with reliable T2E fusion status. Sixty inconsistent samples were used as testing samples for classification performance between 5-ERG-mRPs and fusion prediction tools.

**Fig. 3:**
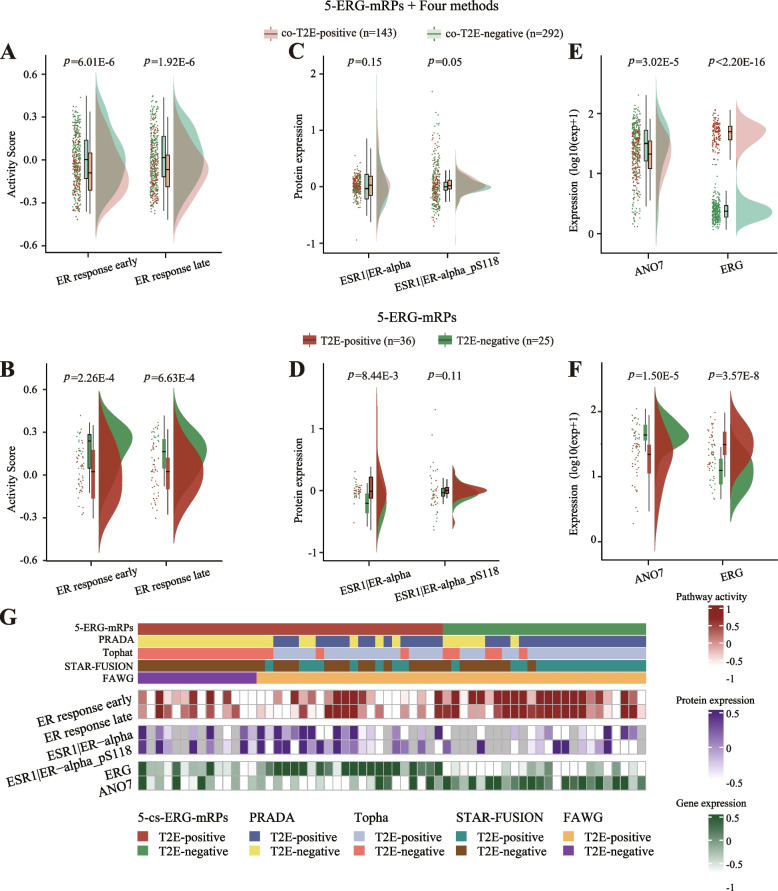
The comparison of performance between 5-ERG-mRPs and fusion prediction tools. Raincloud plots illustrate the distribution of molecular features between TFP and TFN samples grouped by the combination of 5-ERG-mRPs and fusion prediction tools or 5-ERG-mRPs alone, including **A**, **B** the activity score of estrogen response pathway, **C**, **D** the expression of ER-alpha protein, **E**, **F** the expression of *ANO7* and *ERG*. **G** Oncoplot showing the difference of molecular characteristics above between TFP and TFN samples classified by fusion prediction tools. The left shows the label information of prediction tools and molecular features, and grouping annotations are provided at the bottom

Previous study has shown that T2E expression is associated with estrogen receptor (ER) signaling pathway and the expression of ER-alpha protein [17]. By calculating the activity score of HALLMARK pathway “ER response early” and “ER response late” of 435 reference samples using ssGSEA method, we found 143 TFP samples displayed a significantly lower activity score than 292 TFN samples (one-sided Wilcoxon rank-sum test, ER response early, *p* = 6.01E − 6; ER response late, *p* = 1.92E − 6, Fig. 3A). For 60 testing samples, when using the classification result of 5-ERG-mRPs, we obtained similar results that 36 TFP samples showed a decreased ssGSEA score than 25 TFN samples (one-sided Wilcoxon rank-sum, ER response early, *p* = 2.26E − 4; ER response late, *p* = 6.63E − 4, Fig. 3B). For the expression of ER-alpha, a significant result was also observed that the TFP group in reference samples was greater than the TFN group with a clear trend (one-sided Wilcoxon rank-sum, ESR1|ER-alpha, *p* = 0.15; ESR1|ER-alpha_pS118, *p* = 0.05, Fig. 3C). Similar results were also obtained when 5-ERG-mRPs was applied in testing samples (one-sided Wilcoxon rank-sum, ESR1|ER-alpha, *p* = 8.44E − 3; ESR1|ER-alpha_pS118, *p* = 0.11, Fig. 3D). Besides, recent works have reported that the overexpression of *ERG* and the downregulation of *ANO7* were induced by T2E fusion [8, 34]. As expected, for reference samples, TFP samples displayed a significantly decreased *ANO7* expression and elevated *ERG* expression than TFN samples (one-sided Wilcoxon rank-sum, *ANO7*, *p* = 3.02E − 5; *ERG*, *p* < 2.20E − 16, Fig. 3E). According to the classification results of 5-ERG-mRPs for testing samples, TFP samples also showed a significantly lower expression level of *ANO7* and a higher expression of *ERG* than TFN samples (one-sided Wilcoxon rank-sum, *ANO7*, *p* = 1.50E − 5; *ERG*, *p* < 3.57E − 8, Fig. 3F). On the contrary, for these molecular features, no significant or consistent results were observed between the TFP and TFN samples based on the grouping results of fusion prediction tools (Fig. 3G).

### 5-ERG-mRPs owing the predictive ability for overall survival of locally early-stage PC

**Fig. 4:**
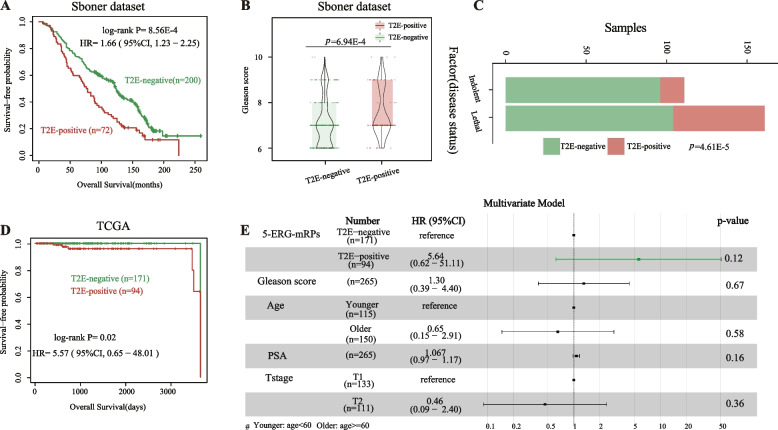
The difference in clinicopathological characteristics between groups based on 5-ERG-mRPs. **A** Kaplan–Meier survival analysis of groups classified by 5-ERG-mRPs in the Sboner dataset. **B** The difference of Gleason score between the TFP and TFN samples classified by 5-ERG-mRPs in the Sboner dataset. **C** The proportions of TFP and TFN tumors in lethal or indolent disease. **D** The Kaplan–Meier curves of OS for samples in the TCGA dataset. **E** Multivariate Cox analyses for 5-ERG-mRPs, Gleason score, age, PSA level, and stage were performed in TCGA. Solid circles represent the HR of death, and the open-ended horizontal lines represent the 95% confidence interval (CI). HR and 95% CIs were generated using multivariate Cox regression models

The clinical outcomes of patients with locally early-stage PC (cT1-2, N0, M0) were highly heterogeneous. Here, survival analysis was conducted to test the predictive ability of 5-ERG-mRPs for the prognosis of these patients. Based on 5-ERG-mRPs, 72 and 200 samples were classified into TFP and TFN groups, respectively. Results showed that patients in TFP group displayed more aggressive than patients in TFN group, including a significantly worse OS (*p* = 8.56E − 4, HR = 1.66, 95% CI: 1.23 − 2.25, Fig. 4A) and a higher Gleason score (one-sided Wilcoxon rank-sum test, *p* = 6.94E − 4, Fig. 4B). Using Fisher’s exact test, a greater proportion of TFP samples in the lethal subtype was observed (*p* = 4.61E − 5, Fig. 4C). Further, we extracted gene expression profile and OS information of 255 patients with locally early-stage PC in dataset TCGA. The 94 patients classified into the TFP group by 5-ERG-mRPs had significantly shorter OS than the 171 patients classified into the TFN group (*p* = 0.02, HR = 5.57, 95% CI: 0.65–48.01, Fig. 4D). Besides, multivariate Cox analysis showed that 5-ERG-mRPs tends to approach formal significance associated with OS of localized patients after adjusting for stage, age, PSA level, and Gleason score (*p* = 0.12, HR = 5.64, 95% CI: 0.62–51.11, Fig. 4E).

### TFP tumor presenting decreased anti-tumor immune infiltration

**Fig. 5:**
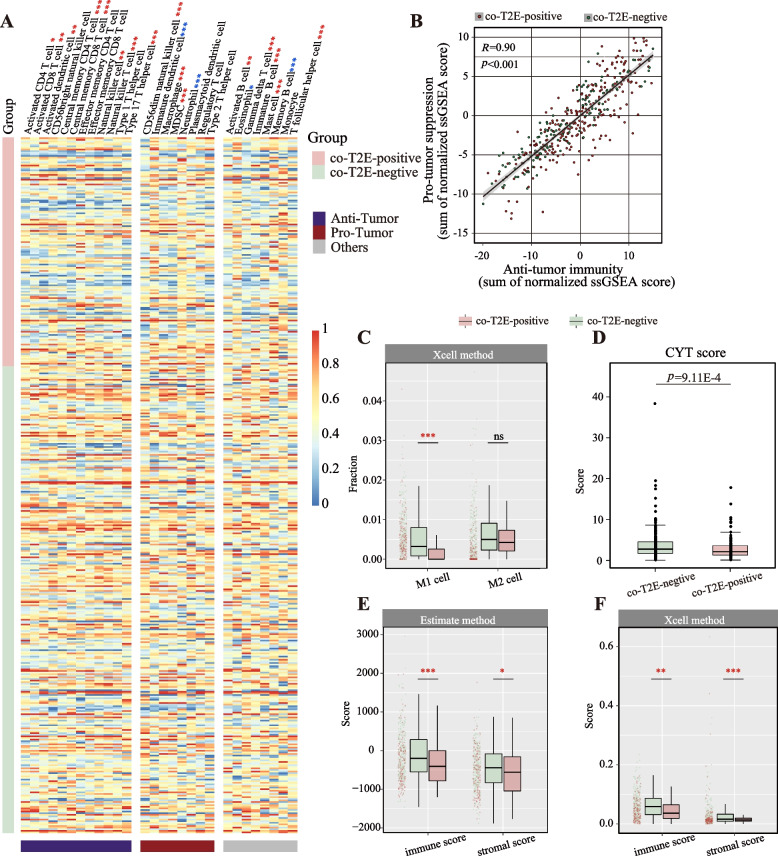
Immune infiltration analysis of TFP tumors. **A** Identifying the relative infiltration of immune cell populations for 435 PC of reference samples in TCGA using ssGSEA method. The relative infiltration of each cell type is normalized into a *z*-score. **B** Relationship between infiltration of cell types executing anti-tumor immunity and cell types executing pro-tumor, immune suppressive functions. *R* coefficient and *p* value were calculated by Pearson’s correlation method. **C** Abundance of M1 and M2 immune cells between the TFP and TFN tumors from the TCGA dataset. **D** Immune cytolytic (CYT) score across 435 PC samples derived from TCGA sequencing data stratified by TFP (red) and TFN (blue) status. Immune score and stromal score computed by **E** estimated method and **F** xCell method between the TFP and TFN samples. *p* values in the heatmap and box plot were determined by one-sided Wilcoxon test. *p < 0.05, **p < 0.01, ***p < 0.001

It is known that tumor aggression is related to tumor immune microenvironments. To determine the relation between T2E fusion status and tumor immune microenvironments in PC, we composed a heatmap to visualize the abundance of 28 infiltrating immune cell populations of 435 reference samples using the ssGSEA method. Results showed that TFP tumors had a lower abundance of immune cell infiltration, especially cells specialized for anti-tumor reactivity (e.g., activated CD4 T cell, activated CD8 T cell, and central memory CD8 T cell, Fig. 5A). Besides, a positive relationship was observed between cells in groups anti-tumor and pro-tumor, suggesting a feedback mechanism that the recruitment of immune suppression cells may be facilitated by anti-tumor inflammation (Fig. 5B).

M1 macrophage and M2 macrophage were two main types of macrophage, which played roles in promoting and inhibiting tumor development, respectively. For cells delivering pro-tumor suppression, compared with TFP tumors, the abundance of macrophage was greater in TFN tumors (Fig. 5A). Furthermore, using xCell, we found the relative fraction of M1 macrophage in TFP tumors was significantly lower than TFN tumors (one-sided Wilcoxon rank-sum test, *p* < 0.001, Fig. 5C). However, no significant difference was obtained for the fraction of M2 macrophage between these two groups (Fig. 5C), indicating the pro-tumor dominant role of M1 cells in TFP tumors. CYT score was an important index for evaluating immune infiltration in anti-tumor [35]. Comparison analysis showed that TFP tumors had a decreased CTY score than TFN tumors (one-sided Wilcoxon rank-sum test,* p* = 9.11E − 4, Fig. 5D). Finally, based on xCell and Estimate, we found that immune score and stromal score were consistently lower in the TFP tumors than in the TFN tumors (one-sided Wilcoxon rank-sum test, Estimate: immune score, *p* < 0.001, stromal score, *p* < 0.05; xCell: immune score, *p* < 0.01, stromal score, *p* < 0.001, Fig. 5E, F).

### Single-cell analysis between TFP and TFN samples

**Fig. 6:**
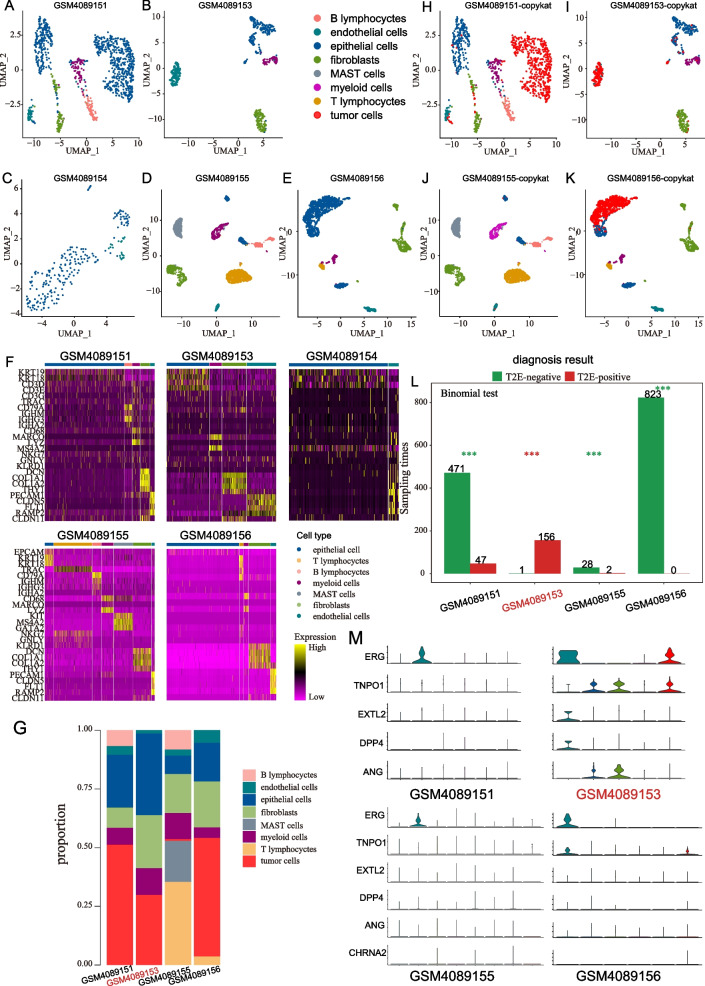
Single-cell analysis between TFP and TFN tumors. **A**–**E** UMAP visualization of 8469 cells among five PC samples. **F** Heatmap of differentially expressed genes (rows) between cells classified into inferred seven cell subsets. Bars on the top of the heatmap indicated the cell type corresponding to those of **A** with selected genes indicated. **G** Distribution of immune-related cells subpopulations in TFP vs TFN tumors. **H**–**K** UMAP of tumor cells identified by the Copycat method. **L** Histogram showed the prediction results of PC samples by 5-ERG-mRPs. The row represented the times that a sample was diagnosed as TFP or TFN sampler by a random method. *p < 0.05, **p < 0.01, ***p < 0.001. **M** Violin plots showing expression of *ERG*, *TNPO1*, *EXTL2*, *DPP4*, and *ANG* in among four samples

We next investigate the difference in tumor microenvironment between TFP and TFN tumors by using single-cell RNA sequencing data. After quality control, 8469 cells from five tissue samples of PC were retained and annotated into seven cell types (see “Materials and methods,” Fig. 6A–E). Canonical cell markers had a distinct expression pattern in the cell types of different clusters (Fig. 6F, Table S1). As shown in Fig. 6G, cell composition presented substantial heterogeneity among different samples. Then, epithelial cells or endothelial cells from four patients were annotated as tumor cells by Copycat method (Fig. 6H–K), and the number of various cells from patients was described in Table S2. By using a random sampling method for tumor cells described in the section method details, sample GSM4089153 and the other three samples (GSM4089151, GSM4089155, and GSM4089156) were divided into TFP and TFN groups by 5-ERG-mRPs, respectively (Fig. 6L). Correspondingly, we also found that *ERG* was expressed only in sample GSM4089153 (Fig. 6M). Besides, cells from sample GSM4089153 did not annotate to MAST cells, T lymphocytes, and B lymphocytes (Fig. 6G), in which MAST cell is related to PC aggressive [36]. On the contrary, T lymphocytes and B lymphocytes from three TFN samples were annotated to CD8 + T cells, T helper 17, T follicular helper, follicular B cell, and immature B cell (Fig. S4A–D), which was consistent with the results in BULK analysis that TFN tumors had a higher anti-tumor immune infiltration than TFP tumors.

### Therapeutic opportunities for TFP tumor

**Fig. 7:**
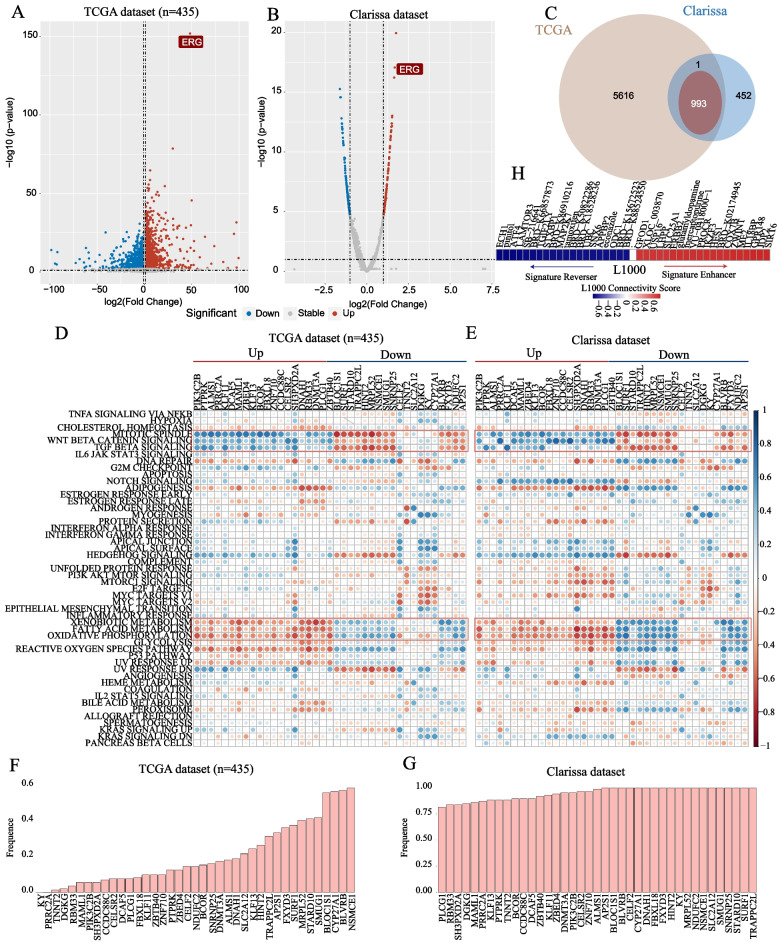
Identification of universal dysregulated genes and potential therapeutic drugs. **A**, **B** Volcano diagram of differentially expressed genes in TCGA and Clarissa datasets. Limma with FDR less than 0.05 was considered to be significant. **C** Venn map of DEGs between TCGA and Clarissa cohorts. **D** Correlation heatmap between 39 universal genes and ssGSEA scores of fifty oncogenic pathways. Red (blue) dots represented a negative (positive) relationship. **E** The dysregulated frequency of 39 genes in TCGA and Clarissa datasets. **F**, **G** The dysregulation frequency of 39 universal DEGs in TCGA and Clarissa datasets. **H** Drug connectivity analysis using alteration-specific transcriptional signatures (CLUE, L1000). Twenty compounds that most strongly reverse or enhance the signature are highlighted

We identified 6610 and 1446 DEGs between TFP and TFN samples from the TCGA (435 reference samples) and Clarissa datasets, respectively (Limma, FDR < 0.01, Fig. 7A, B). The consistency of T2E fusion-related DEGs between the two datasets was 99.90% (993/994), indicating significantly reproducible and consistent transcriptional differences between these two groups (binomial distribution test, *p* < 2.20E − 16, Fig. 7C). Then, based on the algorithm RankComp V1, we calculated the dysregulation frequency of the 993 DEGs. Eight hundred of 993 DEGs were considered universal DEGs, which owned more than 80% of dysregulation frequency in the TCGA or Clarissa datasets. Among them, the expression of 39 universal DEGs was highly associated with the score of fifty oncogenic pathways in these two datasets (Spearman’s rank correlation, |*r*|> 0.6, FDR < 0.01). Besides, as shown in Fig. 7D and E, the expression level of downregulated genes was strongly negatively correlated with the ssGSEA scores of some canonical immune-related pathways, such as Wnt-β-catenin signaling pathway and TGF-β signaling pathway. On the contrary, the upregulated genes were positively related to these pathways. And the activation of Wnt-β-catenin signaling pathway displayed an insufficient T cell infiltration in the tumor microenvironment [37], consistent with the results that TFP tumors presented reduced immune infiltration of activated CD4 T cell and CD8 T cell. The dysregulation frequency of these 39 universal DEGs was also shown in Fig. 7F and G. Finally, we calculated the connectivity score between these 39 DEGs with corresponding transcriptional signatures (L1000 assay). Results showed that compounds such as ECH1, pinitol, and ATF4 have the potential to reverse the transcriptome change caused T2E fusion (Fig. 7H).

## Discussion

In this study, we developed a transcriptional qualitative signature, termed as 5-ERG-mRPs, which could individually demonstrate the T2E fusion status of PC patients. 5-ERG-mRPs was confirmed to be more accurate than *ERG* gene expression alone. Moreover, 5-ERG-mRPs displayed better performance than current popular fusion prediction tools, such as STAR-Fusion. Based on the prediction results of 5-ERG-mRPs, the difference in molecular scars between the TFP and TFN tumors was more distinct. Currently, the OS of locally early-stage PC patients remains controversial. Our results showed that the TFP patients had worse OS than the TFN patients, with a higher Gleason score and a more significant proportion of the aggressive disease. It reminds us that 5-ERG-mRPs, when coupled with other clinical information, might improve the ability to classify patients into different prognostic groups of aggressive and indolent subtypes. It has been suggested that tumor aggression was related to tumor immune microenvironments. We found that the TFP tumors showed a decreased inhibitory immune microenvironment, such as a lower abundance of immune cell infiltration including activated CD4 T cell, activated CD8 T cell, and central memory CD8 T cell, which might lead to the poor OS of TFP patients. The single-cell analysis also showed that cells from the TFP sample did not annotate to anti-tumor-related immune cells. Finally, our data indicated some compounds with the potential to treat TFP tumors.

A few studies tried to identify a transcriptome signature to detect T2E fusion status in PC. Setlur et al. constructed a T2E fusion signature containing 87 genes based on support vector machine; the AUC reached 80% in validation dataset [17]. Similarly, Bismar et al. developed a 10-gene signature, whose accuracy in the validation set ranged from 65 to 83% [18]. The application of these two signatures needs to pre-collect samples for standardized processing; otherwise, the classification results would be affected. Another 100-gene signature constructed by Zhou et al. was only quantitatively scoring samples for T2E fusion without the ability to classify qualitatively [16]. In contrast, the signature we developed here, 5-ERG-mRPs, achieved an overall accuracy of 83%. Besides this, 5-ERG-mRPs have nature advantages [20, 22]: (i) can be applied in different microarray platforms; (ii) can be applied to individual samples without a complex scoring process; and (iii) is resistant to experimental and technical variations. It thus represents a solid alternative to PC management in clinical settings. In addition, green nanomaterials, due to their advantages of biocompatibility, effectiveness, eco-friendliness, low cost, and less toxicity, have been reported being a potential serve as cancer therapeutics or diagnostics agents [38–41].

We also noticed that not only *ERG* but also other genes involved in our classification of T2E fusion status showed a closed relationship with tumor development. For instance, *TNPO1*, one of the genes in 5-ERG-mRPs, played an important role in regulating the nuclear import, self-association, and monoubiquitination of *BAP1* pertinent to oncogenesis [42]. *EXTL2*, a member of the tumor suppressor *EXT* gene family, shaped tumor cell motility and invasion [43]. As a ferroptosis-related gene, *DPP4* was associated with tumor aggression [44]. *CHRNA2* encoded an alpha subunit of the neuronal nicotinic cholinergic receptor whose overexpression promotes the poor prognosis of PC patients [45].

There are a few limitations in this study. Due to the absence of data with PC treated by compounds identified by the cMAP tool, it is necessary to collect related data to validate the therapeutic opportunities for TFP tumors in the future. Besides, when comparing the performance between 5-ERG-mRPs and fusion prediction tools, we were unable to collect PC samples with T2E fusion labels predicted by pathological detection and fusion tools. However, results showed tumors predicted with TFP subtype by 5-ERG-mRPs displayed distinct TFP-related molecular characteristics, including a low activity score of estrogen receptor signaling pathway, a high expression level of ERα protein, *ERG* upregulation, and *ANO7* downregulation. Finally, for the robustness validation of a signature, the more samples the better. For example, Zhang et al. carried out large-scale studies consisting of over 40,000 samples for training and verifying three models to predict the prognosis of PC, and these models showed good discrimination ability [46–48]. Although 1223 samples from three external datasets were used for verifying the signature in this study, we will continuously collect and sequence new data to perform further validation.

## Conclusion

In summary, the identified 5-ERG-mRPs for PC patients could be applied to define the T2E fusion status at the individual level and convenient in a clinical setting. Therefore, 5-ERG-mRPs merits verifying in a prospective clinical trial, which may reduce unnecessary expenses in the detection of T2E fusion status.

## Availability of data and materials

The datasets analyzed during the present study are available from the Gene Expression Omnibus (http://www.ncbi.nlm.nih.gov/geo/), The Cancer Genome Atlas (http://cancergenome.nih.gov/), and Cbioportal (http://www.cbioportal.org/). The accession number of single-cell RNA-seq data of PC is GSE137829.

### Supplementary Information

**Additional file 1:** **Table S1. **List of maker genes in cell annotation. **Table S2. **Annotation results of cells from scRNA-seq samples in single-cell analysis. **Fig. S1. **Five stability indexes (F-stastic, outlier, entropy, CV and MAD) distribution of normal, stable and unstable genes in training dataset. **Fig. S2. **Performance of *ERG* in the training and validation datasets **Fig. S3. **Venn map of diagnosis results between 5-cs-ERG-mRPs and fusion prediction tools for 495 TCGA samples. **Fig. S4. **UMAP of tumor-infiltrating (A-B) T or (C-D) B lymphocytes annotated from samples GSM4089155 or GSM4089156.

## Abbreviations

AUC: Area under the curve
cMAP: Connectivity map
CV: Coefficient of variation
CYT: Cytolytic
DEGs: Differentially expressed genes
IQR: Interquartile range
MAD: Median absolute deviation
OS: Overall survival
PC: Prostate cancer
REOs: Elative expression orderings
TFN: T2E fusion-negative
TFP: T2E fusion-positive
T2E: TMPRSS2-ERG
5-ERG-mRPs: 5 ERG-mRNA pairs

## References

[1] Siegel RL, Miller KD, Wagle NS, Jemal A (2023). Cancer statistics, 2023. CA Cancer J Clin.

[2] Montazersaheb S, Jafari S, Aytemir MD, Ahmadian E, Ardalan M, Zor M, Nasibova A, Monirifar A, Aghdasi S (2023). The synergistic effects of betanin and radiotherapy in a prostate cancer cell line: an in vitro study. Mol Biol Rep.

[3] Mehdizadeh A, Somi MH, Darabi M, Farajnia S, Akbarzadeh A, Montazersaheb S, Yousefi M, Bonyadi M (2017). Liposome-mediated RNA interference delivery against Erk1 and Erk2 does not equally promote chemosensitivity in human hepatocellular carcinoma cell line HepG2. Artif Cells Nanomed Biotechnol.

[4] Valipour B, Abedelahi A, Naderali E, Velaei K, Movassaghpour A, Talebi M, Montazersaheb S, Karimipour M, Darabi M, Chavoshi H, Nozad Charoudeh H (2020). Cord blood stem cell derived CD16(+) NK cells eradicated acute lymphoblastic leukemia cells using with anti-CD47 antibody. Life Sci.

[5] Eftekhari A, Hasanzadeh A, Khalilov R, Hosainzadegan H, Ahmadian E, Eghbal MA (2020). Hepatoprotective role of berberine against paraquat-induced liver toxicity in rat. Environ Sci Pollut Res Int.

[6] Tomlins SA, Rhodes DR, Perner S, Dhanasekaran SM, Mehra R, Sun XW, Varambally S, Cao X, Tchinda J, Kuefer R (2005). Recurrent fusion of TMPRSS2 and ETS transcription factor genes in prostate cancer. Science.

[7] Fernández-Serra A, Rubio-Briones J, García-Casado Z, Solsona E, López-Guerrero JA (2011). Prostate cancer: the revolution of the fusion genes. Actas Urol Esp.

[8] Khosh Kish E, Choudhry M, Gamallat Y, Buharideen SM, D D, Bismar TA. The expression of proto-oncogene ETS-related gene (ERG) plays a central role in the oncogenic mechanism involved in the development and progression of prostate cancer. Int J Mol Sci. 2022;23:4772. https://www.mdpi.com/1422-0067/23/9/4772. 10.3390/ijms23094772 PMC9105369 35563163

[9] Eryilmaz IE, Kordan Y, Vuruskan BA, Kaygısız O, Tunca B, Cecener G (2018). T2E (TMPRSS2-ERG) fusion transcripts are associated with higher levels of AMACR mRNA and a subsequent prostate cancer diagnosis in patients with atypical small acinar proliferation. Gene.

[10] Gong Z, Medeiros LJ, Cortes JE, Zheng L, Khoury JD, Wang W, Tang G, Loghavi S, Luthra R, Yang W (2017). Clinical and prognostic significance of e1a2 BCR-ABL1 transcript subtype in chronic myeloid leukemia. Blood Cancer J.

[11] Kulda V, Topolcan O, Kucera R, Kripnerova M, Srbecka K, Hora M, Hes O, Klecka J, Babuska V, Rousarova M (2016). Prognostic significance of TMPRSS2-ERG fusion gene in prostate cancer. Anticancer Res.

[12] Song C, Chen H (2018). Predictive significance of TMRPSS2-ERG fusion in prostate cancer: a meta-analysis. Cancer Cell Int.

[13] Kobelyatskaya AA, Pudova EA, Snezhkina AV, Fedorova MS, Pavlov VS, Guvatova ZG, Savvateeva MV, Melnikova NV, Dmitriev AA, Trofimov DY, et al. Impact TMPRSS2-ERG molecular subtype on prostate cancer recurrence. Life (Basel). 2021;11:588. https://www.mdpi.com/2075-1729/11/6/588. 10.3390/life11060588 PMC8234735 34205581

[14] Fernández-Serra A, Rubio L, Calatrava A, Rubio-Briones J, Salgado R, Gil-Benso R, Espinet B, García-Casado Z, López-Guerrero JA (2013). Molecular characterization and clinical impact of TMPRSS2-ERG rearrangement on prostate cancer: comparison between FISH and RT-PCR. Biomed Res Int.

[15] Chaux A, Albadine R, Toubaji A, Hicks J, Meeker A, Platz EA, De Marzo AM, Netto GJ (2011). Immunohistochemistry for ERG expression as a surrogate for TMPRSS2-ERG fusion detection in prostatic adenocarcinomas. Am J Surg Pathol.

[16] Zhou E, Zhang B, Zhu K, Schaafsma E, Kumar RD, Cheng C (2020). A TMPRSS2-ERG gene signature predicts prognosis of patients with prostate adenocarcinoma. Clin Transl Med.

[17] Setlur SR, Mertz KD, Hoshida Y, Demichelis F, Lupien M, Perner S, Sboner A, Pawitan Y, Andrén O, Johnson LA (2008). Estrogen-dependent signaling in a molecularly distinct subclass of aggressive prostate cancer. J Natl Cancer Inst.

[18] Bismar TA, Alshalalfa M, Petersen LF, Teng LH, Gerke T, Bakkar A, Al-Mami A, Liu S, Dolph M, Mucci LA, Alhajj R (2014). Interrogation of ERG gene rearrangements in prostate cancer identifies a prognostic 10-gene signature with relevant implication to patients’ clinical outcome. BJU Int.

[19] Guan Q, Yan H, Chen Y, Zheng B, Cai H, He J, Song K, Guo Y, Ao L, Liu H (2018). Quantitative or qualitative transcriptional diagnostic signatures? A case study for colorectal cancer. BMC Genomics.

[20] Liu K, Geng Y, Wang L, Xu H, Zou M, Li Y, Zhao Z, Chen T, Xu F, Sun L (2022). Systematic exploration of the underlying mechanism of gemcitabine resistance in pancreatic adenocarcinoma. Mol Oncol.

[21] Chen T, Yu T, Zhuang S, Geng Y, Xue J, Wang J, Ai L, Chen B, Zhao Z, Li Y (2022). Upregulation of CXCL1 and LY9 contributes to BRCAness in ovarian cancer and mediates response to PARPi and immune checkpoint blockade. Br J Cancer.

[22] Li Y, Zhao Z, Ai L, Wang Y, Liu K, Chen B, Chen T, Zhuang S, Xu H, Zou M (2021). Discovering a qualitative transcriptional signature of homologous recombination defectiveness for prostate cancer.. iScience.

[23] Dong B, Miao J, Wang Y, Luo W, Ji Z, Lai H, Zhang M, Cheng X, Wang J, Fang Y (2020). Single-cell analysis supports a luminal-neuroendocrine transdifferentiation in human prostate cancer. Commun Biol.

[24] Hao Y, Hao S, Andersen-Nissen E, Mauck WM, Zheng S, Butler A, Lee MJ, Wilk AJ, Darby C, Zager M (2021). Integrated analysis of multimodal single-cell data. Cell.

[25] Kim N, Kim HK, Lee K, Hong Y, Cho JH, Choi JW, Lee JI, Suh YL, Ku BM, Eum HH (2020). Single-cell RNA sequencing demonstrates the molecular and cellular reprogramming of metastatic lung adenocarcinoma. Nat Commun.

[26] Gao R, Bai S, Henderson YC, Lin Y, Schalck A, Yan Y, Kumar T, Hu M, Sei E, Davis A (2021). Delineating copy number and clonal substructure in human tumors from single-cell transcriptomes. Nat Biotechnol.

[27] Bhuva DD, Cursons J, Davis MJ (2020). Stable gene expression for normalisation and single-sample scoring. Nucleic Acids Res.

[28] Barbie DA, Tamayo P, Boehm JS, Kim SY, Moody SE, Dunn IF, Schinzel AC, Sandy P, Meylan E, Scholl C (2009). Systematic RNA interference reveals that oncogenic KRAS-driven cancers require TBK1. Nature.

[29] Charoentong P, Finotello F, Angelova M, Mayer C, Efremova M, Rieder D, Hackl H, Trajanoski Z (2017). Pan-cancer immunogenomic analyses reveal genotype-immunophenotype relationships and predictors of response to checkpoint blockade. Cell Rep.

[30] Mandal R, Samstein RM, Lee KW, Havel JJ, Wang H, Krishna C, Sabio EY, Makarov V, Kuo F, Blecua P (2019). Genetic diversity of tumors with mismatch repair deficiency influences anti-PD-1 immunotherapy response. Science.

[31] Bland JM, Altman DG (2004). The logrank test Bmj.

[32] Wang H, Sun Q, Zhao W, Qi L, Gu Y, Li P, Zhang M, Li Y, Liu SL, Guo Z (2015). Individual-level analysis of differential expression of genes and pathways for personalized medicine. Bioinformatics.

[33] Lee M, Lee K, Yu N, Jang I, Choi I, Kim P, Jang YE, Kim B, Kim S, Lee B, et al. ChimerDB 3.0: an enhanced database for fusion genes from cancer transcriptome and literature data mining. Nucleic Acids Res. 2017;45:784–9. 10.1093/nar/gkw1083 PMC5210563 27899563

[34] Marx A, Koopmann L, Höflmayer D, Büscheck F, Hube-Magg C, Steurer S, Eichenauer T, Clauditz TS, Wilczak W, Simon R (2021). Reduced anoctamin 7 (ANO7) expression is a strong and independent predictor of poor prognosis in prostate cancer. Cancer Biol Med.

[35] Hu Q, Nonaka K, Wakiyama H, Miyashita Y, Fujimoto Y, Jogo T, Hokonohara K, Nakanishi R, Hisamatsu Y, Ando K (2021). Cytolytic activity score as a biomarker for antitumor immunity and clinical outcome in patients with gastric cancer. Cancer Med.

[36] Park JW, Lee JK, Phillips JW, Huang P, Cheng D, Huang J, Witte ON (2016). Prostate epithelial cell of origin determines cancer differentiation state in an organoid transformation assay. Proc Natl Acad Sci U S A.

[37] Li X, Xiang Y, Li F, Yin C, Li B, Ke X (2019). WNT/Î^2^-catenin signaling pathway regulating T cell-inflammation in the tumor microenvironment. Front Immunol.

[38] Pearce K, Thipe VC, Henkel RR, Katti KV (2023). Green nanotechnology as an innovative drug delivery approach for Typha capensis and naringenin—new class of phytochemical embedded biocompatible gold nanoparticles in prostate cancer therapy. Journal of Drug Delivery Science and Technology.

[39] Khoobchandani M, Khan A, Katti KK, Thipe VC, Al-Yasiri AY, MohanDoss DKD, Nicholl MB (2021). LugÃ£o AB, Hans CP, Katti KV: **Green nanotechnology of MGF-AuNPs for immunomodulatory intervention in prostate cancer therapy**. Sci Rep.

[40] Barabadi H, Ovais M, Shinwari ZK, Saravanan M (2017). Anti-cancer green bionanomaterials: present status and future prospects. Green Chem Lett Rev.

[41] Sargazi S, Laraib U, Er S, Rahdar A, Hassanisaadi M, Zafar MN, DÃ­ez-Pascual AM, Bilal M. Application of green gold nanoparticles in cancer therapy and diagnosis. Nanomaterials (Basel). 2022;12:1102. https://www.mdpi.com/2079-4991/12/7/1102. 10.3390/nano12071102 PMC9000429 35407220

[42] Yang TJ, Li TN, Huang RS, Pan MY, Lin SY, Lin S, Wu KP, Wang LH, Hsu SD. Tumor suppressor BAP1 nuclear import is governed by transportin-1. J Cell Biol. 2022;221:e202201094. 10.1083/jcb.202201094 PMC9036092 35446349

[43] Marques C (2022). PoÃ§as J, Gomes C, Faria-Ramos I, Reis CA, VivÃ¨s RR, MagalhÃ£es A: **Glycosyltransferases EXTL2 and EXTL3 cellular balance dictates heparan sulfate biosynthesis and shapes gastric cancer cell motility and invasion**. J Biol Chem.

[44] Shi J, Wu P, Sheng L, Sun W, Zhang H (2021). Ferroptosis-related gene signature predicts the prognosis of papillary thyroid carcinoma. Cancer Cell Int.

[45] Fan A, Zhang Y, Cheng J, Li Y, Chen W (2022). A novel prognostic model for prostate cancer based on androgen biosynthetic and catabolic pathways. Front Oncol.

[46] Zhang Z, Cai Q, Wang J, Yao Z, Ji F, Hang Y, Ma J, Jiang H, Yan B, Zhanghuang C (2023). Development and validation of a nomogram to predict cancer-specific survival in nonsurgically treated elderly patients with prostate cancer. Sci Rep.

[47] Zhang Z, Zhanghuang C, Wang J, Tian X, Wu X, Li M, Mi T, Liu J, Jin L, Li M, He D (2022). Development and validation of nomograms to predict cancer-specific survival and overall survival in elderly patients with prostate cancer: a population-based study. Front Oncol.

[48] Zhang Z, Zhanghuang C, Wang J, Mi T, Liu J, Tian X, Jin L, He D (2022). A web-based prediction model for cancer-specific survival of elderly patients undergoing surgery with prostate cancer: a population-based study. Front Public Health.

