# Supplementary material for: Individualized detection of TMPRSS2-ERG fusion status in prostate cancer: a rank-based qualitative transcriptome signature
Source: World J Surg Oncol. 2024 Feb 9;22:49. doi: 10.1186/s12957-024-03314-8 (PMC10854045; doi:10.1186/s12957-024-03314-8)
Supplement: Supplementary file 1 — Additional file 1: Table S1. List of maker genes in cell annotation. Table S2. Annotation results of cells from scRNA-seq samples in single-cell analysis. Fig. S1. Five stability indexes (F-stastic, outlier, entropy, CV and MAD) distribution of normal, stable and unstable genes in training dataset. Fig. S2. Performance of ERG in the training and validation datasets Fig. S3. Venn map of diagnosis results between 5-cs-ERG-mRPs and fusion prediction tools for 495 TCGA samples. Fig. S4. UMAP of tumor-infiltrating (A-B) T or (C-D) B lymphocytes annotated from samples GSM4089155 or GSM4089156. [file 12957_2024_3314_MOESM1_ESM.docx]

**Individualized detection of TMPRSS2-ERG fusion status in prostate cancer: A rank-based qualitative transcriptome signature**

SUPPLEMENTARY MATERIALS

**Table S1** List of maker genes in cell annotation

| Class | Cell types | Maker genes |
| --- | --- | --- |
| 8 cell lineages | Endothelial cell | *PECAM1*, *CLDN5*, *FLT1*, *RAMP2* |
|  | Epithelial cell | *EPCAM*, *KRT19*, *CDH1*, *KRT18* |
|  | Fibroblast | *DCN*, *COL1A1*, *COL1A2*, *THY1* |
|  | Myeloid cell | *CD68*，*MARCO*，*CD16*，*LYZ* |
|  | NK cell | *CD56*, *NKG7*, *GNLY*, *KLRD1* |
|  | T lymphocycte | *CD3D*, *CD3E*, *CD3G*, *TRAC* |
|  | B lymphocycte | *CD79A*, *IGHM*, *IGHG3*, *IGHA2* |
|  | MAST cell | *KIT*, *MS4A2*, *GATA2* |
| T lymphocycte | CD4+ T cell | *Il7R*, *CD4* |
|  | CD8+ T cell | *CD8A*, *CD8B* |
|  | T helper 17 cell | *IRF4*, *CREM*, *NR4A2* |
|  | T helper 1 cell | *STAT4*, *IFNG*, *IL12RB2* |
|  | Gamma delta T cell | *TRDC*, *TRGC2*, *TRGC1* |
|  | T follicular helper cell | *MAF*, *CXCR5*, *PDCD1*, *HAVCR2* |
| B lymphocycte | Immature B cell | *CD19*, *CD20*, *CD34*, *CD38*, *CD45R* |
|  | Activated B cell | CD23 |
|  | Plasma B cell | CD78 |
|  | Memory B cell | *CD80*, *CD84*, *CD86* |
|  | Follicular BCell | *MS4A1*, *HLA-DRA* |
|  | Germinal centers B cell | *STMN1*, *AICDA*, *MKI67*, *BIRC5*, *LMO2*, *BCL2A1* |

**Table S2** Annotation results of cells from scRNA-seq samples in single-cell analysis

| Patient ID | Patient #1 | Patient #2 | Patient #3 | Patient #4 | Patient #5 | Patient #6 |
| --- | --- | --- | --- | --- | --- | --- |
| Cells after QC filtering | 1013 | 13 | 527 | 221 | 5059 | 1636 |
| Number/proportion of tumor cells | 518/0.51 | - | 157/0.30 | - | 30/0.0056 | 823/0.50 |
| Number/proportion of epithelial cells | 227/0.22 | - | 183/0.35 | 200\0.90 | 396/0.08 | 269/0.16 |
| Number/proportion of endothelial cells | 38/0.04 | - | 8/0.02 | 21\0.10 | 135/0.03 | 89/0.05 |
| Number/proportion of fibroblasts | 88/0.09 | - | 119/0.23 | - | 839/0.17 | 320/0.20 |
| Number/proportion of myeloid cells | 73/0.07 | - | 60/0.11 | - | 562/0.11 | 74/0.05 |
| Number/proportion of T lymphocyctes | - | - | - | - | 1787/0.35 | 61/0.04 |
| Number/proportion of B lymphocyctes | 69/0.07 | - | - | - | 417/0.08 | - |
| Number/proportion of MAST cells | - | - | - | - | 893/0.18 | - |

Note：Patient #1, GSM4089151; Patient #2, GSM4089152; Patient #3, GSM4089153; Patient #4, GSM4089154; Patient #5, GSM4089155; Patient #6, GSM4089156


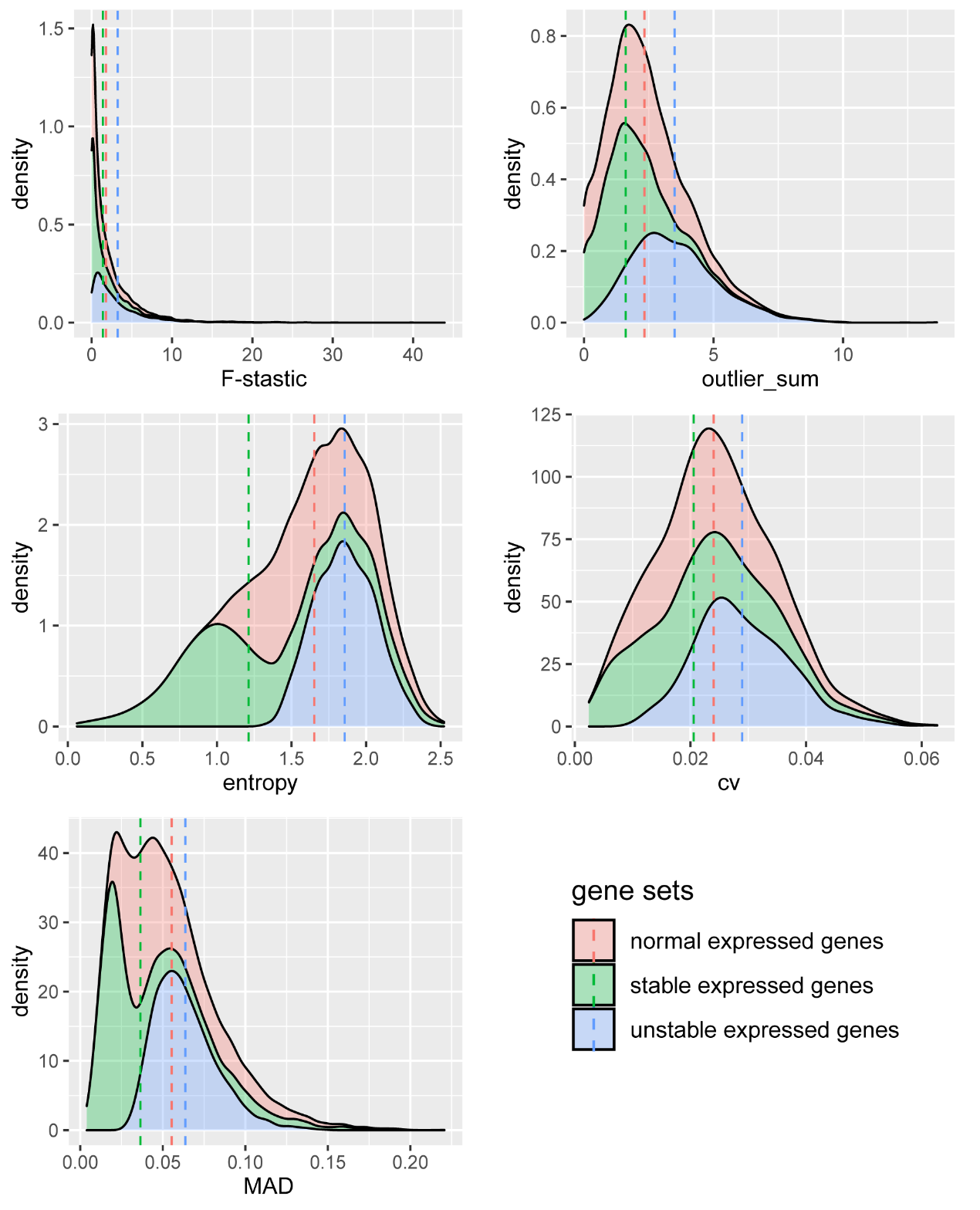


**Figure S1.** Five stability indexes (F-stastic, outlier, entropy, CV and MAD) distribution of normal, stable and unstable genes in training dataset.


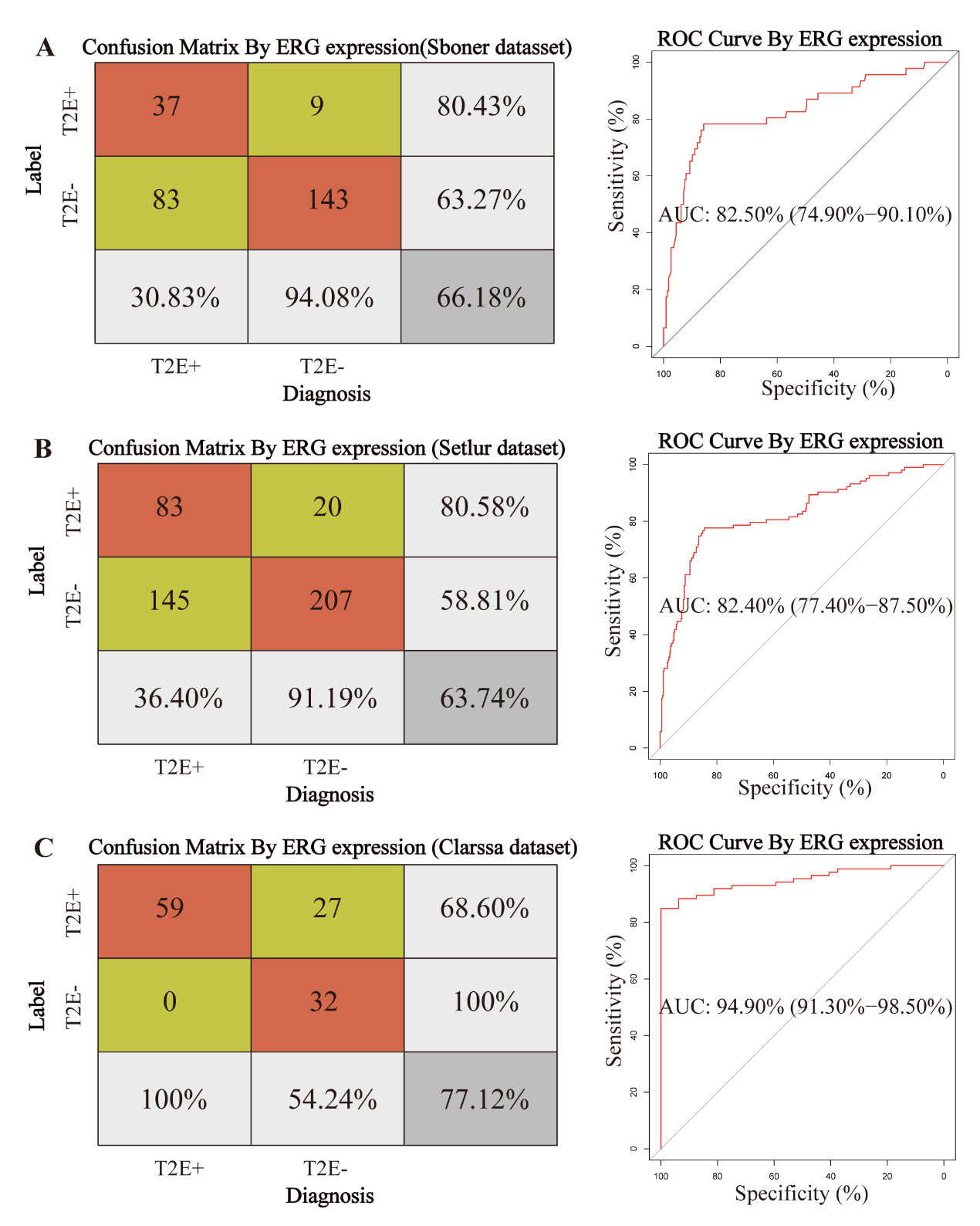


**Figure S2. Performance of *ERG* in the training and validation datasets.**

(A) Confusion matrix and ROC curve for the Sboner dataset by *ERG*. (B) Confusion matrix and ROC curve for the Setlur dataset by *ERG*. (C) Confusion matrix and ROC curve for the Clarissa dataset by *ERG*.


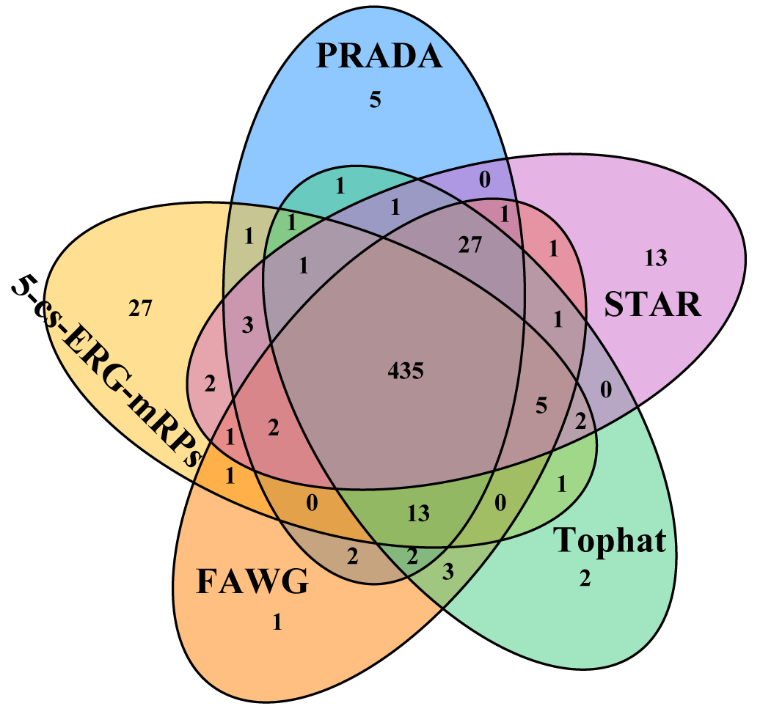


**Figure S3.** Venn map of diagnosis results between 5-cs-ERG-mRPs and fusion prediction tools for 495 TCGA samples.


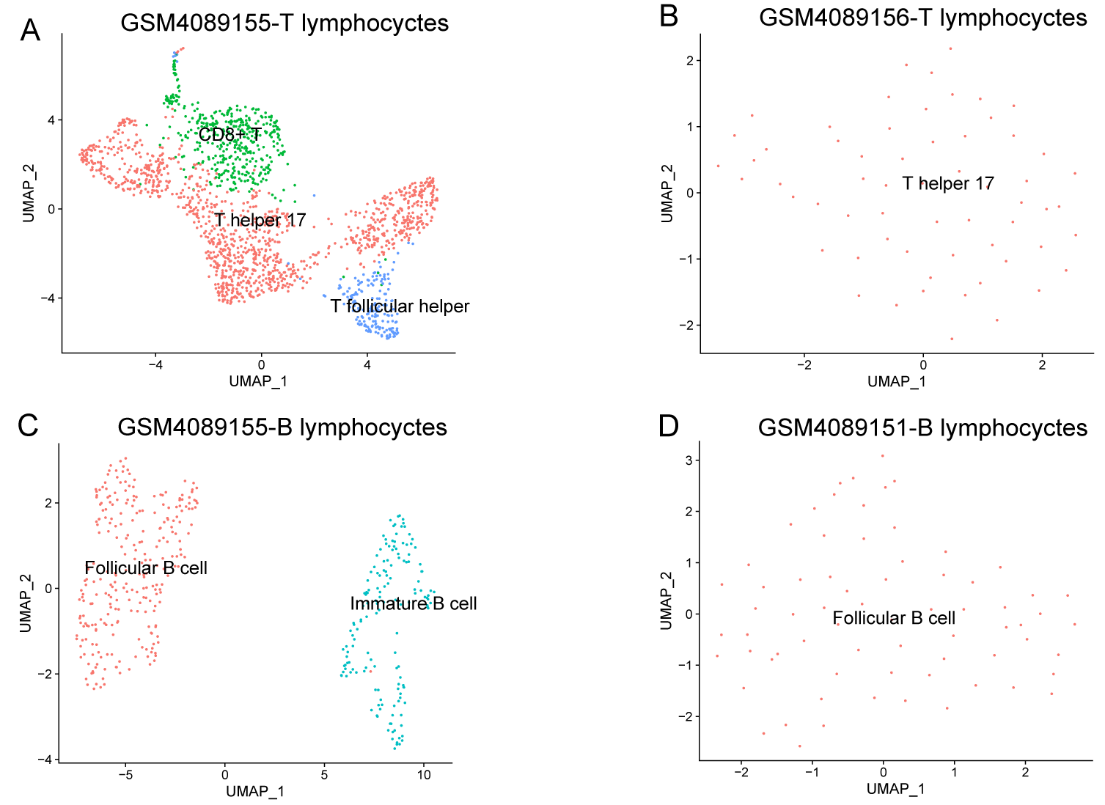


**Figure S4.** UMAP of tumor-infiltrating (A-B) T or (C-D) B lymphocytes annotated from samples GSM4089155 or GSM4089156.
